# Reconceptualizing functional brain connectivity in autism from a developmental perspective

**DOI:** 10.3389/fnhum.2013.00458

**Published:** 2013-08-07

**Authors:** Lucina Q. Uddin, Kaustubh Supekar, Vinod Menon

**Affiliations:** ^1^Department of Psychiatry and Behavioral Sciences, Stanford University School of MedicineStanford, CA, USA; ^2^Program in Neuroscience, Stanford University School of MedicineStanford, CA, USA; ^3^Department of Neurology and Neurological Sciences, Stanford University School of MedicineStanford, CA, USA

**Keywords:** autism spectrum disorders, brain development, functional connectivity, puberty, fMRI

## Abstract

While there is almost universal agreement amongst researchers that autism is associated with alterations in brain connectivity, the precise nature of these alterations continues to be debated. Theoretical and empirical work is beginning to reveal that autism is associated with a complex functional phenotype characterized by both hypo- and hyper-connectivity of large-scale brain systems. It is not yet understood why such conflicting patterns of brain connectivity are observed across different studies, and the factors contributing to these heterogeneous findings have not been identified. Developmental changes in functional connectivity have received inadequate attention to date. We propose that discrepancies between findings of autism related hypo-connectivity and hyper-connectivity might be reconciled by taking developmental changes into account. We review neuroimaging studies of autism, with an emphasis on functional magnetic resonance imaging studies of intrinsic functional connectivity in children, adolescents and adults. The consistent pattern emerging across several studies is that while intrinsic functional connectivity in adolescents and adults with autism is generally reduced compared with age-matched controls, functional connectivity in younger children with the disorder appears to be increased. We suggest that by placing recent empirical findings within a developmental framework, and explicitly characterizing age and pubertal stage in future work, it may be possible to resolve conflicting findings of hypo- and hyper-connectivity in the extant literature and arrive at a more comprehensive understanding of the neurobiology of autism.

## Introduction

Autism spectrum disorder (ASD) is a neurodevelopmental disorder characterized by impaired social interaction and communication, repetitive behaviors, and restricted interests. According to the latest reports, ASD affects nearly 1 in 88 children, and the prevalence continues to grow (Investigators, 2012). The recognition of the increasing prevalence of ASD has placed a mandate on understanding its neurobiological foundations. As highlighted in several articles appearing in this special topic, one of the most well-documented observations in the autism literature is that the brains of individuals with the disorder exhibit aberrant functional connectivity or inter-regional communication (Belmonte et al., 2004). Functional connectivity as measured from functional magnetic resonance imaging (fMRI) data is defined as “temporal correlations between remote neurophysiological events” (Friston, 1994). Functional connectivity is typically measured using one of three approaches: (1) regression analysis using a seed region of interest (Greicius et al., 2003; Fox et al., 2005), (2) full or partial correlation analysis of multiple regions of interest (Ryali et al., 2012), or (3) independent component analysis (ICA) of the entire imaging dataset to identify spatial maps with common temporal profiles (Beckmann and Smith, 2004; Cole et al., 2010). These measures have been used to characterize large-scale networks in the human brain (Bressler and Menon, 2010; Sporns, 2011), and have paved the way for increasingly sophisticated investigations of brain connectivity in ASD (Kennedy and Adolphs, 2012).

Temporal correlations in blood oxygen level dependent (BOLD) fMRI signals are thought to arise from signal propagation and dynamical slowing down of fluctuations in anatomically constrained neural networks (Deco et al., 2013). Consistent with this, empirical studies using human ECoG have shown that slow (<0.1 Hz) spontaneous fluctuations of firing rate and gamma local field potentials are correlated with spontaneous fMRI fluctuations (Nir et al., 2008). Intrinsic functional connectivity measured during resting state fMRI may reflect a history of task-dependent coactivation, and likely serves to organize and coordinate neuronal activity, or might represent dynamic predictions about expected patterns of use (Fox and Raichle, 2007).

Several investigations have reported that functional connectivity between brain regions is weaker in high-functioning ASD, leading to long-distance cortical “under-connectivity” theories of autism (Courchesne and Pierce, 2005; Geschwind and Levitt, 2007; Schipul et al., 2011; Just et al., 2012). However, there is emerging evidence that challenges these models and suggests that functional connectivity between brain regions can be stronger in ASD (Uddin et al., 2013). It is not yet understood why such conflicting patterns of brain connectivity results are observed across different studies, and factors contributing to these heterogeneous findings have not been identified. A more nuanced account capturing patterns of both task-related and intrinsic hypo- and hyper-connectivity observed in autism is essential for characterizing aberrant brain organization in the disorder (Kana et al., 2011; Muller et al., 2011; Vissers et al., 2011). Recent attempts to provide explanations for the discrepant findings in the literature have delineated both methodological issues (Muller et al., 2011) and conceptual issues (Vissers et al., 2011). Here we propose that yet another source of inconsistency exists, namely that developmental changes in functional connectivity have received inadequate attention to date. We posit that discrepancies between findings of autism-related hypo-connectivity and hyper-connectivity might be reconciled by taking developmental stage into account.

The idea that critical periods of plasticity during brain development represent particularly vulnerable stages during which aberrant maturational process can occur is not a new one. In 2003 Rubenstein and Merzenich first introduced the theory that autism may arise from an increased ratio of excitation/inhibition in developing neural systems subserving sensory, mnemonic, social, and emotional processes. Hyperexcitability as a result of this imbalance has been hypothesized to contribute to poorly functionally differentiated and inherently unstable cortex in autism (Rubenstein and Merzenich, 2003). As summarized in a recent review characterizing autism as a critical period disorder, excessive plasticity at the wrong times could result in noisy and unstable processing, yet a brain that lacks appropriate levels of plasticity early in life might remain hyper- or hypo-connected and unresponsive to environmental changes early in life (LeBlanc and Fagiolini, 2011).

One of the earliest signs of autism is enlarged head circumference or macrocephaly (Lainhart et al., 1997). Infants and young children with ASD show signs of early brain overgrowth (Courchesne et al., 2003). Postmortem studies of children with ASD show that they have an overabundance or excess numbers of neurons in the prefrontal cortex (Courchesne et al., 2011). Animal models likewise provide evidence for hyper-connectivity at very early time points in development (Testa-Silva et al., 2011). There is a profound inconsistency between these observations and “under-connectivity” or hypo-connectivity theories that by and large do not account for the possibility of an early phase of neural hyper-connectivity in ASD.

The EEG literature has long reflected an understanding that stabilization and pruning of connections during development plays a central role in the development of cognitive and perceptual functions during critical periods early in life. Uhlhaas and colleagues summarize decades of work to hypothesize that “in ASDs abnormal brain maturation during early prenatal and postnatal periods results in cortical circuits that are unable to support the expression of high-frequency oscillations during infancy. These impaired oscillations might in turn reduce the temporal precision of coordinated firing patterns and thereby disturb activity-dependent circuit selection during further development” (Uhlhaas et al., 2010). These developmental perspectives from animal models and electrophysiological studies should be integrated into the fMRI community, which has struggled to reconcile inconsistent findings with regards to functional brain connectivity in ASD over the past several years.

There has been rapid progress in understanding changes in functional connectivity accompanying typical development with the advent of resting-state fMRI (Uddin et al., 2010). For example, it is now known that subcortical areas are more strongly functionally coupled with primary sensory, association, and paralimbic areas in children, whereas adults show stronger cortico-cortical functional connectivity between paralimbic, limbic, and association areas (Supekar et al., 2009). More generally, several studies have demonstrated that over development, functional brain networks shift from a local anatomical emphasis to a more distributed architecture (Fair et al., 2009; Kelly et al., 2009). It has recently been suggested that motion-related artifacts can have a significant impact on functional connectivity estimates (Power et al., 2012; Van Dijk et al., 2012) in such a way that makes it difficult to study developmental differences. While the appropriate treatment of motion-related artifacts is as yet an unresolved issue in the field (see Satterthwaite et al., 2012, 2013), findings from other imaging modalities including diffusion tensor imaging corroborate functional connectivity findings of increased integrity of long-distance connections with development (Supekar et al., 2010; Uddin et al., 2011). These and other insights from developmental cognitive neuroscience can and should inform theories of atypical development of functional connectivity in autism.

The majority of functional neuroimaging studies of autism have been conducted in adolescents or adults, in part due to practical limitations related to scanning very young children (Yerys et al., 2009). Evidence from these studies of older individuals generally supports the hypo-connectivity theory of autism. However, the lack of available empirical data from younger children with the disorder has made it difficult to test the extent to which the hypo-connectivity theory generalizes to younger age groups. Although calls for data sharing in autism research have been put forth in the past (Belmonte et al., 2008), only recently have large neuroimaging datasets been released. One recent grassroots data sharing initiative (http://fcon_1000.projects.nitrc.org/indi/abide/) has made pre-publication datasets of neuroimaging data collected from individuals between the ages of 6 and 60 available to researchers to facilitate and accelerate the discovery of the functional architecture of the autistic brain (Di Martino et al., 2013a). Still, at this time relatively little has been published addressing the issue of functional brain connectivity in young children with ASD.

The purpose of this review is to (1) summarize the current status of the field by highlighting key findings from studies using fMRI to examine task-related and intrinsic functional connectivity in individuals with ASD across various age groups, (2) reveal critical gaps in the literature which have led to an inconsistent characterization of functional connectivity in ASD, and (3) argue that a developmental perspective can help reconcile some extant contradictory findings, and is necessary for future progress in the field.

## Functional brain connectivity in autism: review

Autism is a disorder with early life onset and variable developmental trajectory (Stefanatos, 2008). Functional neuroimaging studies of young children are thus especially critical for developing accurate models of the underlying neurobiology of the disorder. Thus, it is perhaps surprising that very few fMRI studies have addressed the question of how the brain is functionally organized in childhood ASD, at developmental stages more proximal to the onset of the disorder (Akshoomoff et al., 2002; Amaral, 2010). Below we survey fMRI studies of ASD examining task-based functional connectivity and resting-state functional connectivity with the goal of providing an overview of the existing literature and highlighting the dearth of developmental studies of functional connectivity in ASD.

### Task-based functional connectivity

Task-based functional connectivity measures the synchronization of activation levels between brain regions during the performance of a given cognitive task. Since the initial fMRI reports of hypo-connectivity in autism (Just et al., 2004), task-related reductions in inter-regional brain connectivity during language (Just et al., 2004; Mason et al., 2008; Jones et al., 2010), working memory (Koshino et al., 2005, 2008), mental imagery (Kana et al., 2006), executive functions (Just et al., 2007), cognitive control (Kana et al., 2007; Solomon et al., 2009; Agam et al., 2010), visuomotor coordination (Villalobos et al., 2005) and social cognition (Kleinhans et al., 2008; Kana et al., 2009) have been documented. However, reports of brain hyper-connectivity in ASD also exist in the domains of visuomotor processing (Mizuno et al., 2006; Turner et al., 2006), visual search (Shih et al., 2011), emotion processing (Welchew et al., 2005), memory (Noonan et al., 2009), and language (Shih et al., 2010). These findings are comprehensively reviewed elsewhere (Thai et al., 2009; Schipul et al., 2011; Vissers et al., 2011). A recent review of studies conducted mainly in adults highlights several methodological variables including concatenation of specific task blocks, the use of low-pass filtering, regression of main effects of task, and methods for selecting regions-of-interest that result in considerable heterogeneity between studies with respect to how functional connectivity is conceptualized and analyzed. The authors suggest that such variables may partially account for discrepancies in connectivity results, and that hypo-connectivity findings may be contingent upon these methodological choices (Muller et al., 2011). For example, Muller and colleagues surveyed 32 studies and found that the use of low-pass filtering of fMRI data more often produced results inconsistent with the general under-connectivity theory (Muller et al., 2011).

Recognizing and documenting methodological issues is a critical first step toward synthesizing findings in the “functional connectivity in autism” literature and identifying robust and replicable results. Overall, task-based functional connectivity studies largely support the hypo-connectivity theory, however, the majority of these report results from older adolescents and adults. Additionally, task-based approaches produce results that cannot be easily generalized to other cognitive states, and differences between groups in task performance can make interpretation of hypo- and hyper-connectivity results difficult. The emergence of resting-state fMRI as a means for characterizing the intrinsic functional architecture of the brain, unconfounded by task and behavioral effects, has facilitated data collection from younger typically developing (TD) children and children with ASD (Uddin et al., 2010).

### Resting-state functional connectivity

Since the initial demonstration by Biswal and colleagues that coherent spontaneous low-frequency fluctuations in BOLD signal can be detected within functional systems in the absence of task performance (Biswal et al., 1995), the use of resting-state fMRI in neuroscience has grown exponentially. Applications in clinical neuroscience have been particularly useful, and have provided insights into systems-level cortical and subcortical anomalies of functional connectivity in neurodevelopmental disorders such as attention-deficit/hyperactivity disorder (ADHD, Castellanos and Proal, 2012) and schizophrenia (Yu et al., 2012). Surprisingly, however, there are relatively few published resting-state functional connectivity studies examining individuals with ASD. Extant studies have by and large focused on adolescents or adults with the disorder, with a few notable exceptions.

The earliest published intrinsic functional connectivity study of autism was conducted by Cherkassky and colleagues, who used a seed region-of-interest (ROI) approach to demonstrate functional hypo-connectivity in anterior–posterior connections in adolescents and adults with ASD (Cherkassky et al., 2006). Using an ROI-based approach in another study, Kennedy and colleagues demonstrated disrupted intrinsic connectivity of the default mode network (DMN), but not the dorsal attention network, in a group of adolescents and adults with autism (Kennedy and Courchesne, 2008). Others have replicated this finding of reduced DMN connectivity in adults (Monk et al., 2009), as well as adolescents (Weng et al., 2010) with ASD. Similar findings of decreased functional connectivity of the DMN in adults with ASD have been obtained using data-driven ICA approaches (Assaf et al., 2010), as well as studies combining both seed correlation and ICA (von dem Hagen et al., 2013). Whole-brain connectivity approaches have also provided evidence of hypo-connectivity of social processing-related brain circuits in adolescents with ASD (Gotts et al., 2012), though a recent systematic investigation using both ROI-based and ICA-based analytic approaches found very few examples of functional hypo-connectivity in adults with ASD compared with age-matched control participants (Tyszka et al., 2013). A study of adolescents and adults revealed decreased intrinsic functional connectivity of the insular cortex in high-functioning ASD (Ebisch et al., 2010). As summarized in Table 1, the studies that have reported group differences in the direction of autism-related intrinsic hypo-connectivity were all conducted in either adolescent or adult high-functioning (average or above average IQ) samples. The nascent literature on childhood ASD, in contrast, paints a very different picture.

**Table 1:** **Resting-state functional connectivity MRI studies in ASD**.

**Publication**	**Ages examined**	**Gender and sample size**	**Intrinsic functional connectivity method and results**
Dinstein et al., 2011	ASD: 2.42	29 ASD; 30 TD; gender not reported	Interhemispheric hypo-connectivity between left and right superior temporal gyri and left and right inferior frontal gyri (seed-based during auditory stimulation)
	Range: 1–3.83		
	TD: 2.33		
	Range: 1.08–3.83		
Di Martino et al., 2011	ASD: 10.4 ± 1.7	20 ASD (17 male); 20 TD (14 male)	Hyper-connectivity of striatum with insula and superior temporal gyrus (seed-based)
	TD: 10.9 ± 1.6		
	Range: 7.6–13.5		
Uddin et al., 2013	ASD: 9.96 ± 1.59	20 ASD (16 male); 20 TD (16 male)	Hyper-connectivity within salience, default mode, frontotemporal, motor, and visual networks (ICA)
	Range: 7.52–11.88		
	TD: 9.95 ± 1.60		
	Range: 7.75–12.43		
Lynch et al., 2013	ASD: 9.96 ± 1.59	20 ASD (16 male); 19 TD (15 male)	Hyper-connectivity within default mode network (seed-based)
	TD: 9.88 ± 1.61		
	Range: 7–12		
Washington et al., 2013	ASD: 10.88 ± 2.27	24 ASD (21 male); 24 TD (21 male)	Hyper-connectivity within default mode, visual, and motor networks (ICA), internodal default mode hypo-connectivity (seed-based)
	TD: 10.08 ± 3.17		
	Range: 6–17		
Rudie et al., 2012	ASD: 13.57	38 ASD (32 male); 33 TD (28 male)	Hypo-connectivity within default mode network (seed-based)
	TD: 12.79	
Weng et al., 2010	ASD: 15 ± 1.45 years	16 ASD (15 male); 15 TD (14 male)	Hypo-connectivity within default mode network (seed-based)
	Range: 13–17		
	TD: 16 ± 1.44 years		
	Range: 13–18		
Assaf et al., 2010	ASD: 15.7 ± 3.0	15 ASD (14 male); 15 TD (13 male)	Hypo-connectivity within default mode sub-network (ICA)
	Range: 11–20		
	TD: 17.1 ± 3.6		
	Range: 10–23		
Ebisch et al., 2010	ASD: 15.79 ± 1.93	14 ASD (10 male); 15 TD (13 male)	Hypo-connectivity of insular cortex with amygdala (seed-based)
	TD: 15.95 ± 1.65		
	Range: 12–20		
Gotts et al., 2012	ASD: 16.92 ± 2.66	31 ASD (29 male); 29 TD (28 male)	Whole-brain hypo-connectivity
	TD: 17.86 ± 3.00		
	Range: 12–23		
Cherkassky et al., 2006	ASD: 24 ± 10.6 years	57 ASD (53 male); 57 TD (52 male)	Hypo-connectivity between anterior cingulate and posterior cingulate (seed-based)
	TD: 24 ± 9.0 years		
Kennedy and Courchesne, 2008	ASD: 26.5 ± 12.8 years	12 ASD (12 male); 12 TD (12 male)	Hypo-connectivity within default mode network (seed-based)
	Range: 15.7–52.1		
	TD: 27.5 ± 10.9 years		
	Range: 15.9–45.4		
Monk et al., 2009	ASD: 26 ± 5.93 years	12 ASD (11 male); 12 TD (10 male)	Hypo-connectivity within default mode network (seed-based)
	TD: 27 ± 6.1 years		
Anderson et al., 2011	ASD: 22.4 ± 7.2	53 ASD (53 male); 39 TD (39 male)	Interhemispheric hypo-connectivity between left and right insula and left and right parieto-occipital regions
	Range: 12–42		
	TD: 21.1 ± 6.5		
	Range: 8–34		
Tyszka et al., 2013	ASD: 27.4 ± 2.4	19 ASD (15 male); 20 TD (17 male)	No group differences in whole-brain connectivity (ICA)
	TD: 28.5 ± 2.5		
von dem Hagen et al., 2013	ASD: 30 ± 8:	15 ASD (15 male); 24 TD (24 male)	Hypo-connectivity within default mode network (ICA and seed-based)
	Range: 19–40		
	TD: 25 ± 6		
	Range: 19–36		
Mueller et al., 2013	ASD: 35.5 ± 11.4	12 ASD (9 male); 12 TD (8 male)	Hypo-connectivity within dorsal attention, default mode, and left fronto-parietal network (ICA)
	TD: 33.3 ± 9.0		

*ASD, autism spectrum disorder; TD, typically developing; only studies using resting state fMRI methods are included*.

In a group of children between 7 and 14 years of age, Di Martino and colleagues found that children with ASD exhibit functional hyper-connectivity compared with TD peers. They found increased functional connectivity between striatal subregions and heteromodal association and limbic cortices including insula and superior temporal gyrus (Di Martino et al., 2011). Recently, we demonstrated that children aged 7–12 with autism exhibit hyper-connectivity of several major large-scale brain networks important for cognitive functions. A widely-used method for comparing brain networks between groups is dual regression ICA. Dual regression employs a set of spatial maps derived from the initial group ICA in a linear model fit against each individual fMRI dataset, resulting in matrices describing the temporal dynamics of the corresponding networks for each subject (Beckmann et al., 2005; Filippini et al., 2009). Using this approach, we found that children with ASD exhibited greater functional connectivity than TD children within the DMN, salience, fronto-temporal, motor, and visual networks (Uddin et al., 2013). This somewhat surprising hyper-connectivity result also emerged using complementary analytic approaches and was replicated in several independent datasets (Supekar et al., 2012), and by other groups examining wider age ranges (6–17-year-olds) (Washington et al., 2013). Further, we have found that even within the DMN, hypo- vs. hyper-connectivity results can be observed in children with ASD depending on the precise anatomical location of ROIs within the posterior medial cortex (Lynch et al., 2013). Another recent study of children aged 9–18 found mixed patterns of hypo- and hyper-connectivity between ROIs across the entire brain (Rudie et al., 2013). Rudie and colleagues also report data from graph theoretical analyses demonstrating that while “small worldness” was similar between groups, network level reductions in modularity and clustering as well as shorter characteristic path lengths were observed in children and adolescents with ASD. Some reports of hypo-connectivity between specific ROIs in children with ASD have been published recently (Dinstein et al., 2011; Abrams et al., 2013).

These recent findings raise an important question: If childhood autism is characterized by functional hyper-connectivity, and adults with autism exhibit functional hypo-connectivity, why has the field been slow to examine this important developmental discontinuity? One possible explanation is that the hypo-connectivity theory of ASD has been so dominant that investigators finding contradictory findings have been reluctant to publish their results. This idea has been discussed in a recent Simons Foundation blog (http://sfari.org/news-and-opinion/specials/2013/connectivity/guest-blog-negative-results). A survey of presentations at recent meetings of the International Meeting for Autism Research (IMFAR) and the Organization for Human Brain Mapping (OHBM) suggests that this may in fact be the case. Deen and colleagues conducted ROI-based analyses on data collected from children aged ~13 with ASD and TD control participants. In a poster presented at IMFAR in 2011, they report: “A number of group differences were found in both directions, with no trend toward more differences in the direction of TD>ASD … in the ROI analysis, 19 correlations were stronger in the TD group, while 38 were stronger in the ASD group” (Deen and Pelphrey, 2011). In an HBM poster from the 2012 meeting, You and colleagues reported using a “connectivity degree”—computed by counting, for each voxel, the number of voxels meeting a correlation threshold of *r* > 0.25 inside (local) and outside (distant) its neighborhood defined as a sphere of 14 mm radius (Sepulcre et al., 2010)—to find that degree of functional connectivity was higher in 7–13-year-old children with ASD than TD children (You et al., 2012). These initial findings of functional hyper-connectivity in children with ASD are only now beginning to surface, and may have been initially received with skepticism due to their inconsistency with the hypo-connectivity theory.

## Developmental model of functional brain connectivity in ASD

We propose that the discrepancies between the adult ASD and childhood ASD findings with respect to whole-brain functional connectivity may be reconciled by considering critical developmental factors such as the onset of puberty, which signals the beginning of adolescence and has a major impact on brain structure and function. Puberty typically begins between 9 and 12 years of age, and creates a surge of hormones that trigger rapid physical growth, sexually dimorphic alterations in facial structure, metabolic changes, and several social, behavioral, and emotional changes (Crone and Dahl, 2012). Studies of brain development in animals suggest that the hormonal events surrounding puberty exert significant effects on brain maturation (Cahill, 2006). Relatively few neuroimaging studies have explored the role of puberty in human brain development (Blakemore et al., 2010; Crone and Dahl, 2012; Galvan et al., 2012), though it was noted long ago that measurements of peak gray matter volume coincide with the onset of puberty (Giedd et al., 1999; Blakemore, 2012).

**Figure 1:**
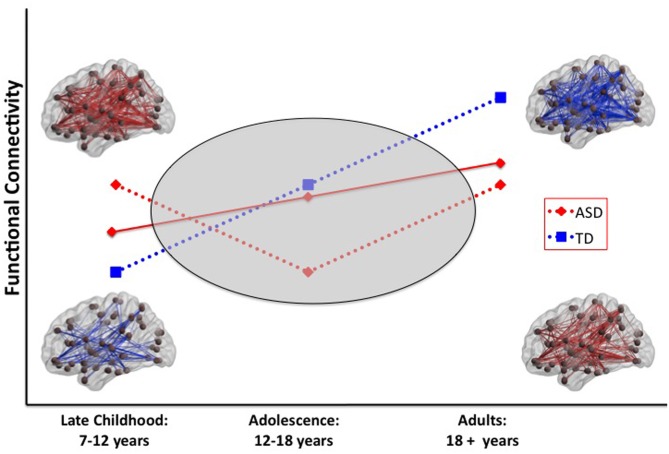
**Schematic model of two scenarios that could explain a developmental shift from intrinsic hyper-to hypo-connectivity in ASD.** In scenario 1 (solid red line), the ASD group shows a less steep developmental increase in functional connectivity over the age span compared with the TD group. In scenario 2 (dashed red line), the ASD group shows anomalous patterns of connectivity across the pubertal period. Resting-state functional connectivity MRI studies provide evidence for widespread hyper-connectivity in children with ASD in contrast to hypo-connectivity observed in adolescents and adults with ASD. To reconcile these findings, it will be necessary to conduct longitudinal studies that span the developmental period surrounding puberty (gray oval). ASD, autism spectrum disorders; TD, typical development.

The age-related discontinuity in the autism neuroimaging literature between findings from children and adults coincides with this developmental period. As summarized in Table 1, studies of children under the age of 12 (presumably predominantly pre-pubertal) find considerable evidence for functional hyper-connectivity in ASD, whereas the studies reporting data collected from adolescents and adults (presumably predominantly post-pubertal) reveal functional hypo-connectivity in ASD. A schematic model of this proposed developmental shift is depicted in Figure 1.

A growing body of literature documents age-related increases in white matter volume (Lenroot and Giedd, 2006), which may be related to increases in long-range functional connectivity from childhood through adolescence and into adulthood (Fair et al., 2008; Kelly et al., 2009). Recent reports of strengthening of structural and functional connectivity with age have shed light on typical developmental processes (Hagmann et al., 2010; Supekar et al., 2010; Uddin et al., 2011). Similar developmental studies of brain connectivity in ASD do not yet exist. In concert with studies of the effects of puberty on typical brain development, this work will help to explain the developmental shift that is suggested by the existing literature of functional connectivity in autism.

## Challenges and gaps in the literature

### Lack of longitudinal data and data from younger participants

The most critical gap in the literature on functional brain connectivity in ASD is the lack of longitudinal studies tracking the same individuals as they progress from pre- to post-pubertal stages of development (Wass, 2011). There are a few longitudinal findings from structural neuroimaging studies spanning the developmental period discussed in this review. One report found significantly greater decreases in gray matter volume in children with autism scanned at two time points (age ~11 and at 30-month follow-up) compared with TD children (Hardan et al., 2009).

While very few studies have examined functional connectivity in young children and toddlers with autism (Dinstein et al., 2011), some have started to use structural measures to examine high-risk infants, including siblings of children with autism. Wolff and colleagues report that infants with ASD showed higher fractional anisotropy (FA) of most fiber tracts at 6 months followed by a slower change over time relative to infants without ASD such that by 24 months of age, the infants with ASD had lower FA values (Wolff et al., 2012). This study suggests that aberrant development of white matter may precede the manifestation of autistic symptoms in the first year of life, and highlights the importance of longitudinal data and data from young children and infants with the disorder.

### Lack of pubertal stage assessment

As highlighted throughout, a potentially informative way of stratifying a sample would be to group individuals by pubertal stage to examine brain maturation as a function of sexual maturity in ASD. Explicit characterization of pubertal stage in research participants can be accomplished in one of several ways. The most widely used tool is the Tanner scale for assessing pubertal development. Tanner staging characterizes individuals along a puberty scale from 1 to 5 on the basis of pubic hair and breast development in females, and pubic hair and genital development in males (Tanner and Whitehouse, 1976). A physical exam carried out by a trained clinician is the typical mode of administration. While there are several limitations to Tanner staging (including ethnic homogeneity of the scale), the test is the current gold standard for puberty assessment. Self-report versions of the scale have also been developed [e.g., the Petersen Developmental Scale (PDS) (Petersen et al., 1988)]. Hormonal assays can also in principle be used to assess pubertal stage, but practical considerations limit their utility Blakemore et al. (2010). Adopting one of these approaches to pubertal assessment when studying adolescents will likely contribute to clarity and interpretability of neuroimaging findings in this population.

### Insufficient characterization of heterogeneity

One significant obstacle to understanding the brain basis of ASD is the fact that the disorder (indeed, disorders) encompasses a wide range of abilities and levels of functioning. Almost no functional brain imaging data is available from individuals who are considered “low-functioning.” Additionally, because of the 4:1 male:female ratio in diagnosis (Werling and Geschwind, 2013), males with the disorder are much more prevalent and therefore receive the majority of attention from researchers. As a consequence, very little is known about gender-specific functional connectivity differences associated with the disorder. It has recently been shown that individuals with variants of the MET gene show differential patterns of resting-state functional connectivity, such that differences between ASD and controls were moderated by genotype (Rudie et al., 2012). This study highlights the important point that studies of disorders characterized by considerable heterogeneity, such as ASD, may need to be particularly mindful of potential genetic differences within their samples.

Reports of relationships between efficiency of functional brain networks and IQ (van den Heuvel et al., 2009) as well as between regional node properties and IQ (Wu et al., 2013) are beginning to emerge. Important directions for future work include assessing interactions between diagnostic category, IQ, and functional connectivity measures. One can speculate that a unique developmental trajectory might exist for children with ASD on the low-functioning end of the spectrum, compared with high-functioning ASD and typical development.

### Direct comparisons between task-based and resting-state functional connectivity

Both task-based and resting-state fMRI have been applied to the study of functional connectivity in ASD. However, to date no empirical work has investigated both types of measures in the same individual. It is clear that methodological choices in both task- and resting-state approaches can affect outcomes in autism neuroimaging studies (Muller et al., 2011). Further, it is increasingly recognized that intrinsic and evoked brain states interact in complex and unpredictable ways (He, 2013). As the field moves closer toward understanding the ways in which task-based and resting-state measures can meaningfully capture brain dynamics, it will continue to inform functional connectivity theories of autism and allow investigators to more confidently predict the conditions under which aberrant brain connectivity in ASD will manifest.

### Relationships between functional and structural connectivity

As the focus of the current review is to summarize findings from the fMRI functional connectivity literature, and structural findings have recently been reviewed elsewhere (Schipul et al., 2011; Vissers et al., 2011), we have included only a limited discussion of the links between structural and functional connectivity here. Relationships between functional and structural connectivity are complex, even in the neurotypical adult brain (Damoiseaux and Greicius, 2009), and these relationships undergo significant changes with development (Supekar et al., 2010; Uddin et al., 2011). In a previously published review of structural connectivity changes in ASD (Vissers et al., 2011), it is noted by the authors that few studies exist simultaneously examining functional and structural changes in ASD. To our knowledge, there are only three reports that do so. In a study by Rudie and colleagues, the authors report that structural connectivity (measured by FA) between the medial prefrontal cortex and posterior cingulate cortex did not show significant differences between ASD and TD children (Rudie et al., 2012). This group has also recently shown that there are no significant differences between groups with respect to structure-function correlations assessed at the whole brain level (Rudie et al., 2013). Finally, it was recently shown that in adults with ASD, reduced functional and structural connectivity can be observed in the right temporo-parietal junction and left frontal lobe (Mueller et al., 2013). The dearth of studies examining structure-function relationships and their development in ASD leaves several open questions that will need to be addressed by future multimodal imaging approaches.

### Assessing whole brain vs. region-specific patterns of functional connectivity

An important area for future work will be to understand functional connectivity abnormalities in ASD at the global level, across the whole brain, as well as in specific functional networks or sets of nodes. There is already evidence to suggest that in children with the disorder, widespread hyper-connectivity can be observed (Supekar et al., 2012; Uddin et al., 2013) alongside both hypo-connectivity (Abrams et al., 2013; Lynch et al., 2013) and hyper-connectivity (Di Martino et al., 2011) between subsets of specific regions. The immediate challenge will be to develop metrics to more systematically assess region-specific and large-scale patterns of connectivity and apply them uniformly to different age groups of individuals with ASD and TD controls.

### Clinical implications: brain-based biomarkers

One of the goals of functional imaging of neurodevelopmental disorders is to quantify brain connectivity in ways that may eventually be used to develop brain-based biomarkers for objectively identifying children with disorders. Anderson and colleagues demonstrate that functional connectivity based classifiers perform more accurately on datasets from younger individuals (<20 years of age) with ASD (Anderson et al., 2011). These findings underscore the importance of understanding age-related changes in functional connectivity in ASD, as they have clear implications for the development of increasingly sophisticated approaches to diagnosis and evaluation of response to treatment. Functional connectivity measures can also aid in understanding unique and shared neural markers in ASD and comorbid conditions such as ADHD (Di Martino et al., 2013b). Our recent demonstration of high levels of classification accuracy based on examination of specific intrinsic large-scale networks in 7–12 year-old children highlights the utility of using data from narrower developmental windows to identify potential biomarkers for the disorder (Uddin et al., 2013).

## Summary and future directions

Inadequate attention to critical age-related developmental stages has impeded our understanding of functional brain connectivity in ASD. Here we have (1) reviewed the emerging literature on intrinsic functional brain connectivity in ASD, (2) identified results of hypo- and hyper-connectivity as being partially attributable to the age of participants examined, and (3) proposed that longitudinal studies examining pre- and post-pubertal individuals with ASD are sorely needed to resolve current controversies regarding the nature of brain connectivity abnormalities in the disorder. A developmental perspective will contribute greatly to future research efforts in autism neuroimaging.

### Conflict of interest statement

The authors declare that the research was conducted in the absence of any commercial or financial relationships that could be construed as a potential conflict of interest.

## Abbreviations

ASD: Autism spectrum disorder
fMRI: Functional magnetic resonance imaging
DMN: Default mode network
ICA: Independent component analysis.
